# Influence of Ongoing Antibiotic Therapy on the Detection of Pathogenic Microorganisms Using Metagenomic Next-Generation Sequencing and Blood Culture in ICU Patients

**DOI:** 10.3390/jcm15124434

**Published:** 2026-06-08

**Authors:** Torsten H. Schroeder, Mamoun Eliwi Alsaffan, Heiner Stäudle, Albion Dervishi

**Affiliations:** 1 Department of Anesthesiology and Intensive Care Medicine, medius KLINIK Nürtingen, Academic Teaching Hospital of the University Hospital Tübingen, 72622 Nürtingen, Germany; 2 Department of Internal and Emergency Medicine, medius KLINIK Nürtingen, Academic Teaching Hospital of the University Hospital Tübingen, 72622 Nürtingen, Germany

**Keywords:** blood culture, intensive care unit (ICU), antimicrobial therapy, bloodstream infection, metagenomic next-generation sequencing

## Abstract

**Background:** Blood cultures often yield negative results in critically ill patients, particularly after antimicrobial therapy has started. Plasma metagenomic next-generation sequencing enables culture-independent pathogen detection, but its diagnostic performance relative to blood cultures, especially under ongoing antibiotic exposure in ICU populations, remains unclear. **Methods:** In this retrospective single-center study, we analyzed adult ICU patients who underwent plasma metagenomic next-generation sequencing testing with paired contemporaneous blood culture between March 2023 and September 2024. Patients were classified according to antibiotic exposure at the time of sampling, and the diagnostic yield and performance of metagenomic next-generation sequencing and blood culture were compared overall and stratified by duration of antibiotic exposure. **Results:** A total of 393 paired metagenomic next-generation sequencing-blood culture samples from 302 ICU patients were analyzed. Blood culture positivity was significantly lower in patients receiving antibiotics at the time of sampling (10.4% vs. 30.4%), whereas metagenomic next-generation sequencing positivity for bacteria remained stable (52.6% vs. 50.8%). With increasing antibiotic exposure, blood culture yield declined sharply, while metagenomic next-generation sequencing detection showed minimal variation. Overall, the concordance of metagenomic next-generation sequencing compared with blood culture as a comparator was 75.3%, with a negative predictive value of 88.0%. Across all subgroups, metagenomic next-generation sequencing demonstrated a higher diagnostic yield than blood culture, with the greatest relative advantage in antibiotic-treated patients. **Conclusions:** In critically ill patients receiving antimicrobial therapy, blood culture diagnostic yield is markedly reduced, whereas plasma metagenomic next-generation sequencing maintains pathogen detection across varying durations of antibiotic exposure. Metagenomic next-generation sequencing represents a valuable complementary diagnostic tool alongside blood cultures in pretreated ICU patients.

## 1. Introduction

Sepsis continues to be the leading cause of morbidity and mortality in the intensive care unit (ICU). Timely and appropriate antimicrobial therapy is a critical determinant of patient survival [1,2,3]. However, blood cultures (BC), the conventional diagnostic standard, often yield negative results in critically ill patients, especially following the initiation of empiric antimicrobial therapy [4]. Metagenomic next-generation sequencing (mNGS), which detects microbial cell-free DNA in blood, has emerged as a culture-independent diagnostic approach. Several studies have reported higher overall positivity rates of mNGS compared with blood cultures (BC) in ICU populations [5,6,7]. In surgical ICU patients, mNGS positivity rates of 72% at onset, compared with 33% for BC, have been reported. Further, mNGS-guided diagnosis led to faster clinical improvement (median 10 vs. 13 days) and more frequent antibiotic de-escalation (21% vs. 11%) [8]. While mNGS offers higher sensitivity and broader pathogen coverage, ongoing antibiotic therapy might affect its diagnostic yield in ICU patients. However, systematic evaluation of how ongoing antimicrobial therapy differentially affects sequencing-based versus culture-based diagnostics remains limited. The primary aim of this study was to compare the effect of ongoing systemic antimicrobial therapy on the diagnostic yield of mNGS versus conventional BC at the time of blood sampling in critically ill ICU patients.

## 2. Methods

### 2.1. Study Design and Population

This retrospective observational study included adult ICU patients (≥18 years) who underwent blood mNGS testing between March 2023 and September 2024 in a tertiary mixed ICU.

**Figure 1:**
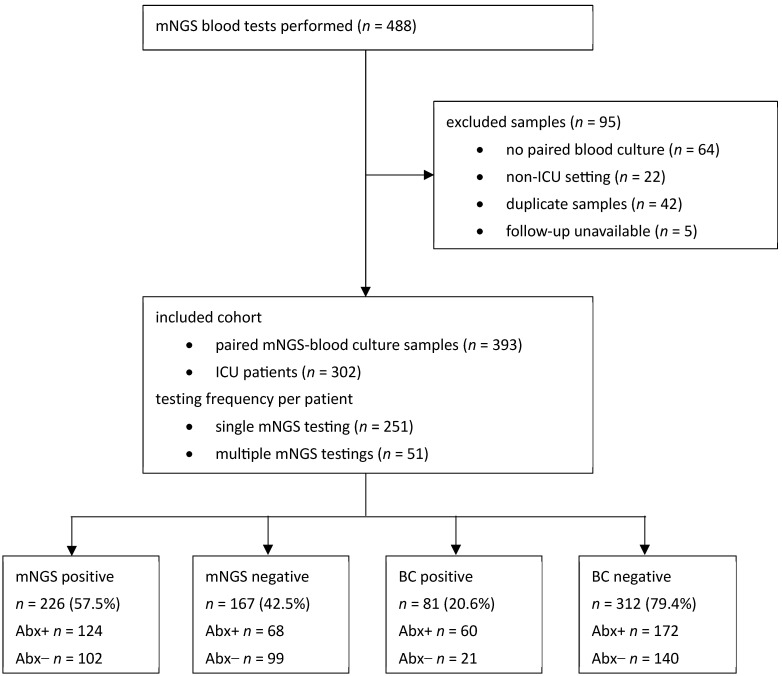
Flow diagram of patient inclusion and distribution of paired mNGS and blood culture samples. *n* = number of samples; mNGS: metagenomic next-generation sequencing; BC: blood culture; ICU: intensive care unit; Abx: Antibiotics.

Blood cultures and mNGS testing were ordered at the clinician’s discretion and, in most cases, drawn concurrently for suspected infection or diagnostic uncertainty. Antibiotic exposure at the time of sampling was not fully controlled because antimicrobial therapy was often initiated prior to ICU admission. Exclusion criteria included missing paired blood cultures, non-ICU treatment, duplicate sampling from the same clinical episode, and incomplete clinical data (Figure 1). Collected variables included patient demographics, admitting service, antimicrobial therapy, its duration at the time of sampling, and blood culture and mNGS results. Fungal and viral mNGS detections were recorded and analyzed descriptively, as they were not predefined study endpoints. This study was conducted in accordance with the principles of the Declaration of Helsinki and applicable national regulations. The study protocol was approved by the Ethics Committee of the Medical Faculty of the University of Tübingen (487/2024-BO2).

### 2.2. Antibiotic Exposure

Patients were classified as Abx+ if systemic antimicrobial therapy was administered for ≥24 h before blood sampling, and as Abx− if no systemic antimicrobial therapy was administered within 24 h prior to sampling. This classification reflects exposure status at the time of sampling and does not imply protocolized timing or indication for antimicrobial initiation. Duration of exposure was recorded in days.

### 2.3. Blood Cultures

Blood cultures were obtained using paired aerobic and anaerobic bottles (BacT/ALERT^®^ SA [Standard Aerobic] and SN [Standard Anaerobic]) and processed according to institutional microbiological protocols.

Organisms were identified by routine biochemical methods, with susceptibility testing performed per standard procedures. Common skin commensals (e.g., coagulase-negative staphylococci) isolated in a single bottle without clinical signs of bloodstream infection were considered potential contaminants and analyzed descriptively but were not excluded from diagnostic yield analyses.

### 2.4. Metagenomic Next-Generation Sequencing

Five to ten milliliters of blood were collected into Streck Cell-Free DNA BCT tubes (Streck Inc., La Vista, NE, USA) and processed in a specialized laboratory (Labor Dr. Wisplinghoff, Cologne, Germany). Cell-free DNA was isolated after two-step centrifugation using the QIAsymphony DSP Circulating DNA Kit (QIAGEN GmbH, Hilden, Germany). Libraries were prepared from 2 ng of cfDNA using the NEXTFLEX® Cell Free DNA-Seq Library Prep Kit 2.0 (Revvity Inc., Waltham, MA, USA) and sequenced on a NextSeq 550 System (Illumina Inc., San Diego, CA, USA) using a NextSeq 500/550 Mid Output Kit. Pathogen identification was performed using the CE-IVD–cleared DISQVER® platform (Noscendo GmbH, Duisburg, Germany) with DISQVER® R6 software, which detects >1100 bacteria, 149 fungi, 78 DNA viruses, and 71 parasites.

### 2.5. Statistical Analysis

Analyses were performed using the R platform (version 4.0). Continuous variables are presented as median (IQR) and compared using the Mann–Whitney U test. Categorical variables are shown as counts (%) and compared by the Chi-square or Fisher’s exact test. Diagnostic performance of mNGS versus blood culture was expressed as sensitivity, specificity, PPV, NPV, and accuracy with 95% CIs. A *p*-value < 0.05 was considered significant. Given the known limitations of blood culture as a reference standard—particularly under antibiotic exposure—all performance metrics are reported as comparative concordance measures between two imperfect tests, rather than absolute diagnostic accuracy. To address potential confounding by disease severity and admission diagnosis, multivariable logistic regression was performed for three outcomes: (1) mNGS positivity, (2) blood culture positivity, and (3) discordant result (mNGS—positive/blood culture-negative). Antibiotic exposure (Abx+ vs. Abx−) was the primary predictor, with SAPS II score and admission diagnosis as covariates. Disease severity was assessed using SAPS II, the validated severity score routinely collected at our institution and widely used in European ICU research.

## 3. Results

### 3.1. Participants and Study Design

A total of 488 blood mNGS tests were screened. After exclusion of 95 samples (no paired blood culture, non-ICU setting, follow-up loss, or duplicate sampling), 393 paired mNGS–blood culture samples from 302 ICU patients were included in the final analysis (Figure 1).

Most patients (*n* = 251, 83%) underwent a single test, whereas 51 patients (17%) underwent serial testing.

### 3.2. Antibiotic Exposure at Sampling

At the time of blood sampling, 132 patients (43.7%) were receiving systemic antibiotics (Abx+), while 170 patients (56.3%) were not (Abx−). Baseline demographic characteristics, comorbidities, and disease severity were comparable between groups (Table 1). Patients in the Abx− group were more frequently admitted from internal medicine services (57% vs. 43%; *p* = 0.02), whereas Abx+ patients more often originated from surgical services (33% vs. 22%; *p* = 0.04). Hospital and ICU lengths of stay were significantly longer in Abx+ patients. Disease severity, assessed by SAPS II, was comparable between groups: median 43.5 (IQR 36–53) in Abx− versus 41.5 (IQR 34–48) in Abx+ patients (*p* = 0.086). To confirm that the observed diagnostic yield differences were not attributable to confounding by severity or clinical indication, multivariable logistic regression was performed. SAPS II was not independently associated with any outcome in the adjusted models (OR 0.99–1.02; *p* = 0.47–0.65). Antibiotic exposure was independently associated with significantly lower blood culture positivity (OR 0.29, 95% CI 0.15–0.54; *p* = 0.0002) and with three times higher odds of a discordant result—defined as mNGS-positive/blood culture-negative—after full adjustment for severity and admission diagnosis (OR 3.11, 95% CI 1.89–5.18; *p* < 0.001). Overall mNGS positivity was not significantly associated with antibiotic exposure after adjustment (OR 1.56, 95% CI 0.96–2.54; *p* = 0.075). These findings confirm that antibiotic therapy selectively suppresses culture-based pathogen detection while mNGS maintains diagnostic yield, independently of disease severity and clinical indication.

### 3.3. Mortality

Mortality outcomes are reported descriptively to characterize cohort acuity and were not analyzed as study endpoints. Overall, in-hospital mortality was 34.4% (104/302; 95% CI, 29.3–40.0), reflecting the high acuity of the cohort. In-hospital mortality did not differ between groups (Abx+: 46/132 [35%] vs. Abx−: 58/170 [34%]; risk ratio 1.02, 95% CI 0.75–1.40; *p* = 0.89). In contrast, ICU mortality was lower among patients receiving antibiotics at the time of blood sampling (Abx+) compared with those not receiving antibiotics (Abx−) (6/132 [4.5%] vs. 29/170 [17%]; *p* < 0.001) (Table 1).

### 3.4. More Positive Results with mNGS Diagnostics in Patients Receiving Antibiotic Therapy

**Figure 2:**
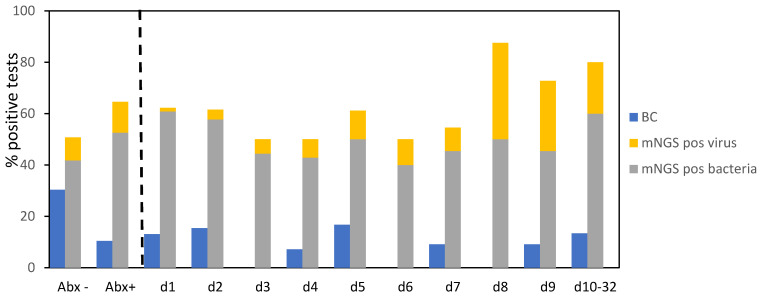
Diagnostic performance of metagenomic next-generation sequencing (mNGS) and blood culture (BC) under antibiotic exposure. Positivity rates for bacterial and viral cfDNA detections in patients with (Abx+) and without (Abx−) antibiotics, as well as trends by duration of antibiotic therapy. Blue bars: positive blood cultures, gray bars: positive mNGS bacteria, yellow bars: positive viral cfDNA detections. Per-stratum sample sizes: Abx− *n* = 201; Abx+ (overall) *n* = 192; d1 *n* = 46, d2 *n* = 26, d3 *n* = 18, d4 *n* = 14, d5 *n* = 18, d6 *n* = 10, d7 *n* = 11, d8 *n* = 8, d9 *n* = 11, d10–32 *n* = 30. Day-level analyses are exploratory and hypothesis-generating; no correction for multiple comparisons was applied.

We compared microbiological positivity between patients with and without antibiotic therapy and across different exposure durations (Figure 2). Blood culture positivity was markedly reduced with antibiotic use (10.4% vs. 30.4%), whereas mNGS detection remained stable (52.6% vs. 50.8%). With longer antibiotic exposure, mNGS positivity showed minimal variation (42.9–60.0%), while blood culture rates declined sharply, often to zero (0–16.7%).

Compared with blood culture, mNGS showed a concordance-based positivity rate of 75.3% (95% CI, 64.9–83.5) and a negative predictive value of 88.0% (95% CI, 82.2–92.1). These metrics reflect concordance between two imperfect tests rather than absolute diagnostic accuracy. Without antibiotics, mNGS concordance-based specificity and positive predictive value (PPV) were 60.0% (95% CI, 51.7–67.7) and 45.1% (95% CI, 35.8–54.8), respectively. Under antibiotics, these fell to 36.6% (95% CI, 29.8–44.0) and 12.1% (95% CI, 7.5–19.0), while the NPV increased to 92.6% (95% CI, 83.9–96.8) (Supplementary Table S1).

Across all subgroups, mNGS demonstrated a higher diagnostic yield than blood culture, with the largest relative advantage in antibiotic-treated patients. Overall, mNGS positivity did not differ by antibiotic exposure (*p* = 0.816), whereas blood culture positivity was significantly lower in treated patients (*p* < 0.001).

### 3.5. More Bacteria Detected by mNGS Compared with Blood Culture

**Figure 3:**
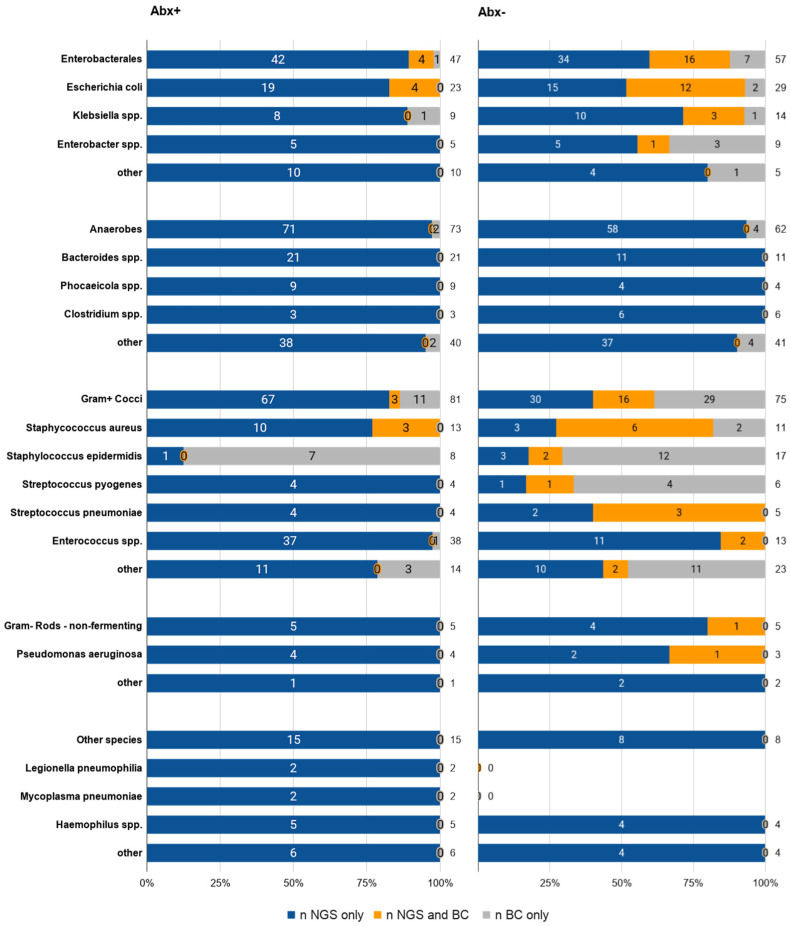
Comparative distribution of bacteria detected by metagenomic next-generation sequencing (mNGS) and conventional blood culture (BC), stratified by antibiotic exposure at the time of sampling. Overlap between mNGS and BC detections, categorized as mNGS-only (blue), mNGS+ BC+ (yellow), or BC-only (gray). Total number of bacteria identified in antibiotic-treated (Abx+) and antibiotic-free (Abx−) samples displayed on the right side of the bars.

To compare bacterial detection across methods, organisms were grouped into five categories: Enterobacterales, anaerobes, Gram-positive cocci, Gram-negative rods (including non-fermenters), and others. Samples were further stratified by antibiotic exposure at the time of sampling (Abx− vs. Abx+) (Figure 3).

In Abx− patients, 102 mNGS-positive samples detected 167 bacteria, versus 71 from 61 positive blood cultures (BC). In Abx+ patients, 124 mNGS-positive samples detected 203 bacteria, versus 21 from 20 positive BC. In the Abx+ cohort, most organisms were mNGS-exclusive, with minimal mNGS-BC overlap and few BC-only detections. Conversely, Abx− patients showed more shared detections and frequent BC-only findings. Prior antibiotics markedly reduced BC diagnostic yield but left mNGS sensitivity largely intact.

Mainly, *Staphylococcus epidermidis* grew exclusively on plates from Abx+ BC samples (*n* = 7) as well as Abx− samples (*n* = 14). Other potential contaminants retrieved from BC samples included *Staphylococcus haemolyticus* (*n* = 3). Nevertheless, four *Streptococcus pyogenes* from patients without antibiotic therapy were not identified by mNGS, compared to five *Streptococcus pyogenes* not identified by BC but by mNGS. In contrast, anaerobes were rarely detected by BC samples, compared to mNGS (129 individual organisms across 43 cases vs. 6 by BC). To assess the clinical relevance of mNGS-exclusive anaerobe detections, we reviewed the clinical context of all 43 cases in which anaerobic organisms were identified by mNGS but not by blood culture. The majority occurred in patients with intra-abdominal pathology (*n* = 28, 65.1%), including bowel perforation, peritonitis, anastomotic dehiscence, and gangrenous cholecystitis—conditions in which translocation of gut anaerobic flora into the bloodstream is a well-established pathophysiological mechanism. The remaining cases involved respiratory infection, including aspiration pneumonia (*n* = 6, 14%), skin and soft tissue infection (*n* = 5, 11.6%), and four cases with an unclear focus (*n* = 4, 9.3%). Anaerobic blood cultures were positive in only 8 of these 43 cases (18.6%), consistent with the known low sensitivity of conventional culture for obligate anaerobes. Therapy modification was indicated in 17 of 43 anaerobe-positive cases (39.5%) and was implemented in the majority, supporting the clinical actionability of mNGS anaerobe detection in this cohort.

Among the four cases with mNGS-exclusive detection of atypical pathogens-*Legionella pneumophila* (*n* = 2) and *Mycoplasma pneumoniae* (*n* = 2)-all presented with respiratory infection and negative or non-contributory blood cultures in three of four cases. Antimicrobial therapy was modified in all four cases (100%) following the mNGS result: treatment was de-escalated from broad-spectrum beta-lactam regimens to targeted agents (levofloxacin or doxycycline) in each instance. In one case, the *Mycoplasma pneumoniae* detection was independently corroborated by serology, providing external validation of the mNGS finding. All four patients survived to hospital discharge. These findings directly support the clinical utility of mNGS for detecting atypical pathogens that are not identifiable by conventional blood culture and demonstrate that mNGS results informed and modified antimicrobial management in this cohort.

### 3.6. Viral cfDNA and Fungi in mNGS Samples

DNA-virus positivity was higher in antibiotic-exposed samples (Abx+: 12.0%) compared with antibiotic-naive samples (Abx−: 9.0%). With increasing duration of antibiotic exposure, the percentage increased to 37.5%. mNGS detected 93 viral cfDNA signals, most frequently *Epstein–Barr virus* (*n* = 40), *Herpes simples virus-1* (*n* = 19), *Cytomegalovirus* (*n* = 13), and human herpes virus-6B (*n* = 11). Fungal cfDNA was found in three samples (*Candida glabrata*, *Candida albicans*, *Aspergillus fumigatus*). One case yielded *Saccharomyces cerevisiae*; in the clinical context, this finding was considered more likely to represent probiotic-associated detection or colonization rather than invasive fungal infection.

## 4. Discussion

The key finding of this study is that ongoing antibiotic therapy markedly reduces the diagnostic yield of blood cultures in critically ill patients, whereas mNGS maintains stable pathogen detection even with prolonged antimicrobial exposure. This divergence highlights the limitations of culture-based diagnostics in pretreated ICU populations and supports the role of mNGS as a complementary diagnostic modality in pretreated ICU patients. Multivariable logistic regression confirmed that this divergence is independent of disease severity and clinical indication: antibiotic exposure was associated with a 71% reduction in blood culture positivity (OR 0.29, 95% CI 0.15–0.54; *p* = 0.0002) and a three-fold increase in discordant results—where mNGS detected a pathogen missed by blood culture—after adjustment for SAPS II and admission diagnosis (OR 3.11, 95% CI 1.89–5.18; *p* < 0.001). SAPS II was not independently associated with any outcome (*p* = 0.47–0.65), confirming that severity did not confound the findings. These results provide the first adjusted evidence that antibiotic therapy selectively suppresses culture-based detection while mNGS maintains diagnostic yield, independently of how sick the patient is or what they are admitted for.

Consistent with prior ICU studies, blood cultures underperformed once antibiotics were initiated, while mNGS retained its diagnostic yield. In cohorts with prolonged antibiotic exposure, mNGS positivity rates of 65–75% have been reported, compared with 10–13% for concomitant blood cultures, emphasizing diagnostic complementarity rather than interchangeability [9].

Similar paired analyses showed mNGS positivity rates of 70–75%, while concomitant cultures were positive in 10–12% of cases, again favoring mNGS for pathogen detection, including fastidious organisms and viruses [10].

Importantly for the ICU, mNGS performance did not appear to be impaired by prior antimicrobials in our study, as reported previously [11]. BC positivity dropped to 10.4% in patients receiving antibiotic therapy, while mNGS testing remained positive in 52.6% of patients on antibiotics (64.6% including DNA viruses), independent of the duration of antibiotic therapy. Overall, mNGS positivity was unaffected by antibiotic exposure, although detection of DNA viruses increased with longer treatment duration.

Although not a study endpoint, ICU mortality was significantly lower among patients receiving antibiotics at the time of blood sampling (Abx+) compared with those not receiving antibiotics. This could reflect that patients in the Abx+ group were already receiving appropriate antibiotic treatment for their infection, whereas the Abx− group may have included patients with new, fulminant sepsis who decompensated rapidly before or shortly after treatment was initiated.

The lower specificity and positive predictive value, especially under antibiotic therapy, likely reflect limitations of BC as the reference standard. Importantly, blood culture was used as the reference comparator in this study; however, its known limited sensitivity—particularly under antibiotic exposure—may lead to underestimation of true mNGS-positive cases and consequently to an apparent reduction in specificity and positive predictive value [4,5,11]. Therefore, diagnostic performance metrics of mNGS should be interpreted relative to blood culture rather than as an absolute measure of accuracy.

Our results are in line with previously published data and reinforce the potential role of mNGS as a complementary diagnostic tool to BC, particularly for patients already receiving antimicrobial therapy [5,12,13].

Parallel testing of blood culture and mNGS remains essential for several reasons. First, mNGS can identify pathogens missed under antibiotic pressure, broaden the diagnostic spectrum to include viral and fungal organisms, and inform clinical interpretation in complex ICU cases [9,14].

However, plasma-based mNGS cannot reliably distinguish active infection from transient DNAemia, colonization, or residual microbial DNA after antimicrobial therapy, requiring careful clinical interpretation. Occasional detection of *Streptococcus pyogenes* by blood culture but not by mNGS has also been reported previously and likely reflects low circulating DNA burden, sampling variability, or technical limitations in cfDNA recovery rather than systematic assay failure [15].

Viral cfDNA signals were detected in 82 cases, predominantly among patients with intra-abdominal infections (28%), respiratory infections (24%), and non-infectious primary critical illness (16%). Blood cultures were negative in 83% of viral-positive cases, and 66% had an ICU stay ≥14 days—confirming a high-acuity, prolonged-illness phenotype in whom herpesvirus reactivation is clinically expected. The most frequently detected viruses—EBV, HSV-1, CMV, and HHV-6B—are precisely those known to undergo stress-related reactivation in critically ill patients [16]. The observed increase in viral cfDNA detection with prolonged antibiotic exposure (up to 37.5%) is consistent with this mechanism, as extended antimicrobial therapy progressively impairs immune surveillance and facilitates latent herpesvirus reactivation. The predominance of EBV (*n* = 40) and HSV-1 (*n* = 19) mirrors patterns reported in mixed ICU populations [17].

These viral cfDNA signals must be interpreted in a clinical context: in patients with short ICU stays and no immunocompromise, they most likely represent subclinical reactivation, whereas in those with prolonged critical illness, organ failure, or immunosuppression, they may warrant targeted antiviral evaluation. mNGS thus captures virome information entirely inaccessible to conventional blood culture, and its interpretation requires integration with clinical and laboratory findings.

Second, multidrug-resistant organisms are currently defined by antimicrobial susceptibility testing of viable blood culture isolates, a capability not yet provided by mNGS. In our cohort, only three multidrug-resistant *Escherichia coli* isolates were identified by both methods among 81 positive blood cultures.

Positive BC results often showed possible contaminants (e.g., *Staphylococcus epidermidis*) when obtained from patients on antibiotics, while the patterns of microorganisms in mNGS results of ICU patients on antibiotics remained similar to those observed in patients without antibiotics. Notably, 32 of the 81 (39.5%) positive BC results were deemed likely contaminants (e.g., coagulase-negative staphylococci, others, time-to-positivity, number of positive samples, clinical appearance). The high rate of likely contaminants in blood cultures further complicates direct comparison and highlights the interpretative limitations of culture-based diagnostics. mNGS can help distinguish between contamination and infection in these patients.

A formal cost-effectiveness analysis was beyond the scope of this study and warrants a dedicated prospective evaluation.

## 5. Limitations

This study has several limitations. First, its retrospective, single-center design may limit generalizability and preclude causal inference. Second, although antibiotic therapy followed standardized institutional protocols once severe infection was suspected, the initial decision, timing, and choice of antimicrobial therapy were frequently made prior to ICU admission in the emergency department, operating room, or referring wards. As a result, antibiotic exposure at the time of sampling was not fully controlled, introducing heterogeneity consistent with real-world ICU practice. Although the antibiotic class was documented for all cases, formal multivariable adjustment for antibiotic class was not performed because antibiotic selection was strongly associated with infection type and clinical indication. This represents a limitation of the retrospective design and should be addressed in future prospective studies.

In addition, plasma-based mNGS detects circulating microbial DNA and cannot reliably distinguish active bloodstream infection from residual DNAemia or transient translocation, requiring careful clinical interpretation. As mNGS testing was performed at the clinician’s discretion, the cohort may be enriched for complex cases, limiting generalizability. As some patients contributed multiple samples, within-patient correlation cannot be excluded, and results should be interpreted at the sample level.

## 6. Conclusions

In critically ill patients receiving antibiotic therapy, blood culture diagnostic yield is markedly reduced, whereas blood mNGS maintains pathogen detection across varying durations of antimicrobial exposure. mNGS, therefore, represents a valuable complementary diagnostic tool in pretreated ICU patients. Prospective studies are needed to define the optimal diagnostic integration of mNGS alongside conventional blood cultures in critically ill patients and to determine whether mNGS-guided diagnostics translate into changes in therapy and improved clinical outcomes.

## Figures and Tables

**Table 1:** Patient characteristics at the time of mNGS sampling. Values are median (IQR) or *n* (%). *p*-values derived from Wilcoxon rank-sum (continuous) or Fisher’s exact (categorical) tests. Abx = antibiotics; ICU = intensive care unit. SAPS II = Simplified Acute Physiology Score II. SAPS II is the standard severity metric used and was comparable between groups (*p* = 0.086).

Patients (*n* = 302)	Abx Positive (*n* = 132)	Abx Negative (*n* = 170)	*p* Value
Age (median, IQR)	73 (62, 82)	75 (65, 82)	0.2
Male *n* = 188 (62%)	76 (58%)	112 (66%)	0.2
Female *n* = 114 (38%)	56 (42%)	58 (34%)	
Preexisting disease			
Hypertension *n* (%)	76 (57.6%)	78 (45.9%)	0.12
Cardiovascular disease *n* (%)	92 (69.7%)	103 (60.6%)	0.2
Pulmonary disease *n* (%)	59 (44.7%)	80 (47.1%)	0.3
Renal disease *n* (%)	54 (40.9%)	63 (37.1%)	0.7
History of stroke *n* (%)	17 (12.9%)	15 (8.8%)	0.4
Diabetes mellitus *n* (%)	42 (31.8%)	54 (31.8%)	0.3
Medical specialty			
Internal medicine *n* (%)	57 (43.2%)	97 (57.1%)	0.02
Surgery *n* (%)	75 (56.8%)	73 (42.9%)	
General	44 (33.3%)	38 (22.4%)	0.04
Orthopedic	12 (9.1%)	20 (11.8%)	0.57
Vascular	9 (6.3%)	5 (3.0%)	0.17
Plastic	4 (5.0%)	4 (2.4%)	0.73
Spinal	6 (7.5%)	6 (3.5%)	0.76
SAPS II	41.5 (34–48)	43.5 (36–53)	0.086
Hospital stay (days, median [IQR])	20 (10, 37)	17 (9, 31)	0.036
ICU stay (days, median [IQR])	8 (4, 17)	5 (3, 12)	0.005
In-ICU mortality *n* (%)	6 (4.5%)	29 (17%)	<0.001
In-hospital mortality *n* (%)	46 (35%)	58 (34%)	>0.9

## Data Availability

The datasets generated and/or analyzed during the current study are available from the corresponding author on reasonable request. The manuscript does not contain any individual person’s identifiable data.

## Supplementary Materials

The following supporting information can be downloaded at: https://www.mdpi.com/article/10.3390/jcm15124434/s1, Table S1. Diagnostic performance of mNGS versus blood culture expressed as sensitivity, specificity, positive predictive value, negative predictive value, accuracy, and area under the curve in percent with 95% confidence interval (CI).
