# Supplementary material for: Influence of Ongoing Antibiotic Therapy on the Detection of Pathogenic Microorganisms Using Metagenomic Next-Generation Sequencing and Blood Culture in ICU Patients
Source: J Clin Med. 2026 Jun 8;15(12):4434. doi: 10.3390/jcm15124434 (PMC13301659; doi:10.3390/jcm15124434)
Supplement: Supplementary file 1 [file jcm-15-04434-s001.zip › jcm-4281350-supplementary.pdf]

	All Cases (With & Without Antibiotics)		No antibiotics during blood collection			Antibiotics during blood collection		
n=393	BC positive, *n*=81	BC negative, *n*=312	*n*=201	BC positive, *n*=61	BC negative, *n*=140	*n*=192	BC positive, *n*=20	BC negative, *n*=172
NGS positive, *n*=226	61	20	*n*=102	46	15	*n*=124	15	5
NGS negative, *n*=167	165	147	*n*=99	56	84	*n*=68	109	63
Sensitivity (%), (95% CI)	75.30%	(64.9% - 83.49%)		75.40%	(63.3% - 84.5%)		75%	(53.1% - 88.8%)
Specificity (%), (95% CI)	47.10%	(41.6% - 52.7%)		60%	(51.7% - 67.7%)		36.60%	(29.8% - 44%)
PPV (%), (95% CI)	27%	(21.6% - 33.1%)		45.10%	(35.8% - 54.8%)		12.10%	(7.5% - 19%)
NPV (%), (95% CI)	88%	(82.2%- 92.1%)		84.80%	(76.5% - 90.6%)		92.60%	(83.9% - 96.8%)
Accuracy (%), (95% CI)	52.90%	(48%- 57.8%)		64.70%	(57.8% - 71%)		40.60%	(33.9% - 47.7%)
AUC (%), (95% CI)	61.20%	(55.7% - 66.7%)		67.70%	(60.9 - 74.5%)		55.80%	(45.4% - 66.2%)
n=393	NGS positive, *n*=226	NGS negative, *n*=167	*n*=201	NGS positive, *n*=102	NGS negative, *n*=99	*n*=192	NGS positive, *n*=124	NGS negative, *n*=68
BC positive, *n*=81	61	165	*n*=61	46	56	*n*=20	15	109
BC negative, *n*=312	20	147	*n*=140	15	84	*n*=172	5	63
Sensitivity (%), (95% CI)	27%	(21.6%-33.1%)		45.10%	(35.8% - 54.8%)		12.10%	(7.5% - 19%)
Specificity (%), (95% CI)	88%	(82.2%- 92.1%)		84.80%	(76.5% - 90.6%)		92.60%	(83.9% - 96.8%)
PPV (%), (95% CI)	75.30%	(64.9% - 83.49%)		75.40%	(63.3% - 84.5%)		75%	(53.1% - 88.8%)
NPV (%), (95% CI)	47.10%	(41.6% - 52.7%)		60%	(51.7% - 67.7%)		36.60%	(29.8% - 44%)
Accuracy (%), (95% CI)	52.90%	(48%- 57.8%)		64.70%	(57.8% - 71%)		40.60%	(33.9% - 47.7%)
AUC (%), (95% CI)	57.50%	(53.7% - 66.7%)		65%	(59% - 71%)		52.40%	(48.1% - 56.6%)

Suppl. Table S1. Diagnostic performance of mNGS versus blood culture expressed as sensitivity, specificity, positive predictive value, negative predictive value, accuracy, and area under the curve in percent with 95% confidence interval (CI).
